# Re-evaluation of diagnostic parameters is crucial for obtaining accurate data on idiopathic pulmonary fibrosis

**DOI:** 10.1186/s12890-015-0074-3

**Published:** 2015-08-19

**Authors:** Jaana Kaunisto, K. Kelloniemi, E. Sutinen, U. Hodgson, A. Piilonen, R. Kaarteenaho, R. Mäkitaro, M. Purokivi, E. Lappi-Blanco, S. Saarelainen, H. Kankaanranta, A. Mursu, M. Kanervisto, E-R. Salomaa, M. Myllärniemi

**Affiliations:** Division of Medicine, Pulmonary Diseases, Turku University Hospital, University of Turku, Turku, Finland; Department of Pulmonary Diseases and Clinical Allergology, University of Turku, Turku, Finland; HUS Medical Imaging Center, Radiology, University of Helsinki and Helsinki University Hospital, Helsinki, Finland; University of Helsinki and Helsinki University Central Hospital, Heart and Lung Center, Helsinki, Finland; Department of Internal Medicine, Respiratory Diseases, University of Oulu, Oulu, Finland; Medical Research Center Oulu, Respiratory Research Unit, Oulu University Hospital, Oulu, Finland; Unit of Medicine and Clinical Research, Pulmonary Division, University of Eastern Finland, Kuopio, Finland; Center for Medicine and Clinical Research, Division of Respiratory Medicine, Kuopio University Hospital, Kuopio, Finland; Department of Internal Medicine, Respiratory Diseases, Institute of Clinical Medicine, University of Oulu, Oulu, Finland; Department of Pathology, Oulu University Hospital and Oulu University, Oulu, Finland; Medical Research Center Oulu, Oulu, Finland; Department of Respiratory Medicine, Tampere University Hospital, Tampere, Finland; Department of Respiratory Medicine, Seinäjoki Central Hospital, Seinäjoki, Finland; Department of Respiratory Medicine, University of Tampere, Tampere, Finland; City Hospital of Oulu and Hoitoketju Coronaria Oy, Oulu, Finland; University of Tampere, School of Health Sciences, Tampere, Finland; University of Turku, Turku, Finland; University of Helsinki and Helsinki University Hospital, Heart and Lung Center and Transplantation laboratory, Helsinki, Finland

**Keywords:** Idiopathic pulmonary fibrosis, Register, Epidemiology

## Abstract

**Background:**

The FinnishIPF registry is a prospective, longitudinal national registry study on the epidemiology of idiopathic pulmonary fibrosis (IPF). It was designed to describe the characteristics, management and prognosis of prevalent and incident IPF patients. The study was initiated in 2012.

**Methods:**

We present here results limited to five university hospitals. Patients with IPF were screened from hospital registries using ICD-10 diagnosis codes J84.1 and J84.9. All patients who gave informed consent were included and evaluated using novel diagnostic criteria. Point prevalence on the 31^st^ of December in 2012 was calculated using the reported population in each university hospital city as the denominator.

**Results:**

Patients with ICD-10 codes J84.1 and J84.9 yielded a heterogeneous group – on the basis of patient records assessed by pulmonologists only 20–30 % of the cases were IPF. After clinical, radiological and histological re-evaluation 111 of 123 (90 %) of patients fulfilled the clinical criteria of IPF. The estimated prevalence of IPF was 8.6 cases/100 000. 60.4 % were men. Forty four percent of the patients were never-smokers. At diagnosis, the patients’ mean age was 73.5 years and mean FVC was 80.4 % and DLCO 57.3 % of predicted.

**Conclusions:**

Our results suggest that hospital registries are inaccurate for epidemiological studies unless patients are carefully re-evaluated. IPF is diagnosed in Finland at a stage when lung function is still quite well preserved. Smoking in patients with IPF was less common than in previous reports.

## Background

According to previous epidemiological studies, the prevalence of idiopathic pulmonary fibrosis (IPF) varies greatly depending on the methods and diagnostic criteria used [1]. As novel treatment options for IPF are emerging [2, 3], accurate epidemiological data on IPF is needed. There are several ongoing national and international projects that aim to determine the epidemiology of IPF [4–7].

The updated ATS/ERS/JRS/ALAT recommendations on the diagnosis and management of IPF [8] emphasize the role of high-resolution computed tomography (HRCT). So far, very few epidemiological studies exist where patients have been re-evaluated based on the novel guidelines, and the ones that do exist, are not geographically extensive [9]. Our study was designed to obtain accurate data on the epidemiology and demographics of carefully re-evaluated IPF patients from five university cities in Finland.

Mortality in IPF is high, but recent studies suggest, that the severity of disease at diagnosis has an effect on mortality – all-cause mortality is relatively low in patients with mild to moderate lung impairment [10, 11]. Delayed access to tertiary care defined as the time from the onset of the dyspnea to the date of the initial evaluation at a tertiary care center is associated with a higher mortality rate in IPF, independent of disease severity [12]. Although these results are not surprising, they accentuate the importance of early diagnosis in IPF.

Cigarette smoking is identified as a risk factor for IPF [13]. In a recent report of a Danish cohort 81 % of IPF patients were current or ex- smokers [14]. It has been previously postulated, that smoking would present a survival benefit in IPF patients [15], but this result has not been confirmed in later studies.

The FinnishIPF study was initiated to assess the characteristics, diagnostic accuracy, treatment, exacerbations and survival of patients with IPF. Enrolling patients and collecting follow-up data are on continuing basis. In this report, we present epidemiological results on systematically collected, re-evaluated IPF patients. Re-evaluation was performed by a multidisciplinary team of pulmonary physicians, radiologists and pathologists in all of the five university hospitals in Finland.

## Methods

### Patient recruitment and data collection

**Fig. 1:**
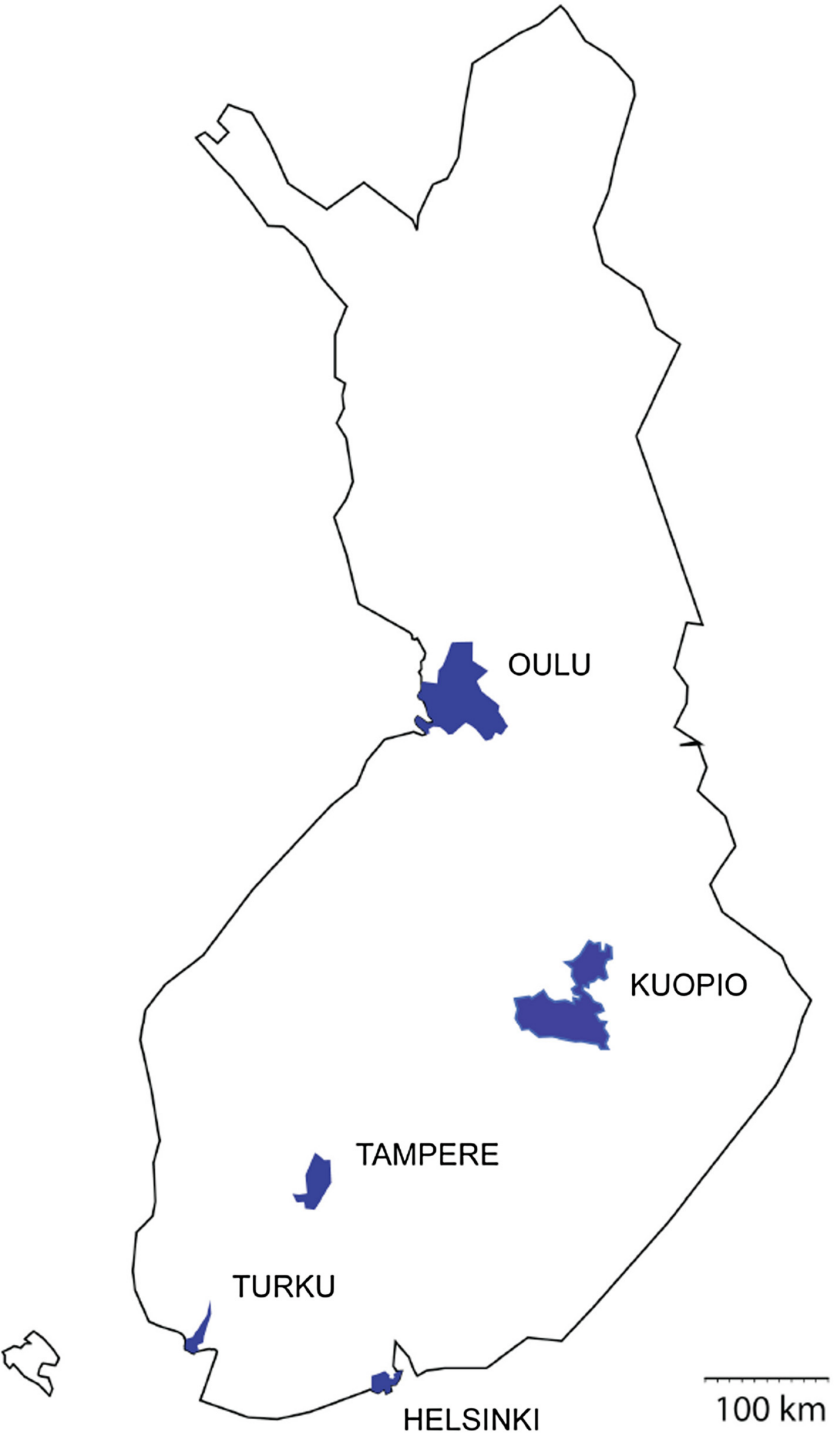
The geographical location and areas of the five university hospital cities in Finland where the cohort was collected and their population. The number of people living in these cities was 1.29 million and it represented 24 % of the total population in Finland

In order to evaluate systematically the nationwide prevalence of IPF in different geographical areas, we narrowed the study population to five university hospital cities and their populations at the end of the year 2012. The university hospitals represent tertiary hospitals, the most specialized level of public health care. All IPF patients who gave informed consent and lived during 2012 in the university hospital cities Helsinki, Turku, Tampere, Kuopio, or Oulu (see Fig. 1 for geographical location) were included. In Finland, patients are referred to specialist centers according to their living address and practically all IPF patients are initially evaluated at the public health care system.

The patient registries of five university hospitals were screened for the ICD-10 diagnosis code J84.1 (other interstitial pulmonary diseases with fibrosis) and J84.9 (interstitial pulmonary disease). The diagnostic criteria of the ATS/ERS statement 2011 [8] were used. An experienced pulmonary physician re-evaluated the patients’ data by reading through the patient charts. 70–80 % of patients’ diagnoses did not meet the clinical criteria of idiopathic pulmonary fibrosis. The disqualified cases were other idiopathic interstitial pneumonias (IIP), most commonly NSIPs (non-specific interstitial pneumonia), fibrotic conditions linked to connective tissue diseases or other types of pulmonary fibrosis with known etiology (e.g. asbestosis). Only patients with confirmed IPF diagnosis were asked to give informed consent. 65–89 % (average 76 %) of the re-evaluated IPF patients gave consent. The patient data was added to a secure, electronic registry database. At baseline, data on demographics, risk factors and comorbidities were collected (Table 1). At follow-up visits lung function parameters as well as events such as hospitalizations, disease exacerbations, changes in medication were recorded (Table 1). The lung function values were evaluated using the Finnish reference values [16]. The dates of death were confirmed from an electronic population registry. The immediate and underlying cause of death was collected from the death certificates (an up-to-date registry kept by Statistics Finland). Missing data was not computed as we had most data on all patients available. Only 4 patients did not have spirometry and only 2 patients did not have smoking history data.

**Table 1:** Parameters collected to the FinnishIPF registry

Basic information	ID, gender, date of birth
	Height, weight, body mass index
	Smoking (pack years), occupation, exposures
	Medical history, chronic illnesses
	Medication
Diagnostic information	Symptoms, date of onset
	Date of diagnosis
	FVC(L), FVC % of predicted, FEV1(L), FEV1 % of predicted, DLCO/VA % of predicted and DLCO % of predicted
	Chest X-ray
	High resolution computed tomography of the lung (HRCT)
	Biopsies, bronchoalveolar lavage samples, laboratory findings
	6-minute walk test (meters)
	Familial or sporadic IPF
Follow-up information	Changes in condition
	FVC(L), FVC % of predicted, FEV1(L), FEV1 % of predicted, DLCO % of predicted, DLCO/VA % of predicted
	Laboratory findings
	High resolution computed tomography of the lung (HRCT)
	6-minute walk test (meters)
	Changes in medication
	Hospitalization
	Date of lung transplantation
	Date of death, cause of death

### Re-evaluation of radiological findings

The baseline CT scans were centrally re-evaluated by one experienced chest radiologist (over 20 years of experience) and a radiology resident. Most of the scans were carried out prior to 2010 with a time range from 2003 to 2012. HRCT scans were evaluated on all of the 123 patients with the exception of one patient, whose evaluation was based on spiral CT. The quality of the spiral CT was considered diagnostic and the patient was therefore not excluded from the study. The scanning protocols and used CT scanners in each center varied. The obtained slice thickness was mainly 1–1.25 mm and the slices were captured in 10–40 mm intervals, one volume HRCT was performed. In 21 patients, expiratory HRCT scans were available. In 13 patients, HRCT had been performed in both supine and prone positions. Six patients had HRCT scan only in the prone position, but they also had a supine helical CT with i.v. contrast media. In two patients, a helical scan with i.v. contrast media, from which HRCT reconstructions were calculated, was available. Altogether 35 patients had a helical scan, 30 with and five without i.v. contrast media. Of the 122 HRCT scans image quality was satisfactory in 119 cases and poor in three, mostly due to motion artifacts. Still, these three HRCT scans with inferior image quality were considered diagnostic and the patients were not excluded from the study. The scans were categorized into three groups: 1) usual interstitial pneumonia (UIP) pattern, 2) possible UIP pattern, and 3) inconsistent with UIP pattern [8]. The presence of a hiatal hernia was assessed when suitable radiological data was available, uncertain findings were not included. Honeycombing as well as other HRCT features were evaluated according to Fleischner Society’s glossary of terms for thoracic imaging [17].

### Re-evaluation of surgical lung biopsies

Surgical lung biopsy was performed in 27 (22 %) patients. Three samples were not available for re-evaluation and in each of these cases initial evaluation was considered to be valid. The biopsies were re-evaluated according to the most recent ATS/ERS guidelines by two experienced pathologists (RK, EL-B) of whom RK is additionally a pulmonologist. The samples were categorized into four groups 1) UIP, 2) probable UIP, 3) possible UIP or 4) not UIP.

### Ethical considerations

Approval from the Helsinki University Hospital ethical committee was obtained, and this approval was accepted by ethical committees of the remaining four university hospitals. The National Institute of Health and Welfare gave authorization to screen patients from all Finnish hospitals which have a unit of respiratory medicine with the consent of the physician in charge. Patients who gave their informed consent were included. Patients who had died during the year 2012 before giving consent were not included.

### Statistical methods

The statistical analysis was carried out by experienced researcher (MK) using SPSS 20.0 for Windows (SPSS™ Illinois, Chicago^©^). Percentages and mean values (95 % CI) were used to describe the data. Kruskall-Wallis test and *X*^2^ test (*p* < 0.05) were used to compare the differences between the groups.

## Results

### Subject characteristics

Table 2 shows the characteristics of the IPF patients who met the used IPF criteria. 60.4 % were men. The mean age of patients at diagnosis was 73.5 years. Only 8.1 % of patients were current smokers. The most common symptoms at the onset of the disease were dry cough (48 of the 103 patients, 46.6 % who had symptom data available) and dyspnea (46/103, 44.7 %). Eleven patients (10.6 %) were asymptomatic at diagnosis. The mean delay from the onset of symptoms to the date of diagnosis was 1.9 years (range 0–16 years, SD 2.9). The mean FVC at diagnosis was 80.4 % of predicted (Table 2). The distribution of FVC and DLCO (% predicted) of the IPF patients in five university hospital cities did not differ from one another (one-way ANOVA).

**Table 2:** Characteristics of the study population (*N* = 111) mean (95 % CI) or %

	Mean or %	95 % CI	Number of observations
Age	73.5	71.8 – 75.2	111
Gender (%)			111
Men	60.4		
Women	39.3		
BMI^a^ (kg/m^2^)	28.1	27.2 – 29.0	98
Smoking (%)			109
Never	44.1		
Former	45.9		
Current	8.1		
FVC^b^ % pred.	80.4	77.4 – 83.3	107
FVC (L)	2.9	2.7 – 3.1	107
DLCO/VA^c^ % pred.	78.4	75.3 – 81.5	104
DLCO % pred.	57.3	54.4 – 60.2	104

Definitions of abbreviations: ^a^ *BMI* Body Mass Index; ^b^ *FVC* Forced Vital Capacity, ^c^ *DLCO(VA)* Diffusing Capacity of Carbon Monoxide (divided by Alveolar Volume)

### Radiological and histopathological re-evaluation

Table 3 shows the classification of patients according to HRCT re-evaluation. Patients with a HRCT finding inconsistent with UIP pattern whose diagnosis was not confirmed by a surgical lung biopsy were dropped out of the final study population (*N* = 11). The patients having a radiological possible UIP pattern who were not biopsied (*N* = 10) were still included in the study, as all patients were evaluated in a multidisciplinary meeting to have IPF. From the 11 patients who were excluded from the initial cohort due to radiological re-evaluation, eight had a HRCT pattern more typical of NSIP than UIP. One looked more like sarcoidosis and one patient who had undergone irradiation due to breast cancer was considered having radiation-induced fibrosis. One patient had an undetermined interstitial lung disease, possible exposures were looked for but none were found in the registry data. Radiological honeycombing was initially seen in 80 % of cases and in the re-evaluation 76 % of cases. The presence or absence of honeycombing was not mentioned in 11 % of the initial readings despite most of these scans were read as typical UIP. The initial readings were mostly prior to the new ATS/ERS criteria, which can explain why honeycombing was not always mentioned.

**Table 3:** Results of the radiological and histopathological re-evaluation, showing the number of biopsy confirmed cases and number of patients included in the study. There was no significant difference in the diagnostic accuracy between the five university hospitals. After multidisciplinary evaluation altogether 111 patients were considered to have IPF

		Histopathological finding					
Radiological finding	*n* (%)	N:O of biopsies	UIP	Probable UIP	Possible UIP	Not UIP	IPF in multidisciplinary evaluation (%)
UIP pattern	87 (70.7)	10	7	1	1	1	87
Possible UIP pattern	18 (14.6)	7	6	1			18
Inconsistent with UIP pattern	18 (14.6)	7	4	1	1	1	6
Total	123	24	17	3	2	2	111 (90.2)

A group of patients classified as having a radiologically typical UIP pattern (*N* = 87) had gone through surgical lung biopsy (*N* = 12) of which 10 samples were available for histopathological re-evaluation. One patient with “not UIP pattern” in sparse lung biopsy was, however, considered as IPF after multidisciplinary evaluation and follow-up. Eighteen HRCT scans were classified as “inconsistent with UIP”. In this group seven lung biopsies were performed; histopathological re-evaluation confirmed four UIP patterns, one probable UIP pattern, one possible UIP and one pattern with “not UIP” (Table 3). After a final multidisciplinary evaluation only the one patient with “not UIP” pattern in surgical lung biopsy was excluded from the cohort. Thus, the final study population used for estimating disease prevalence consisted of 111 confident IPF patients from the university hospital cities (Fig. 1). The results of the radiological and histological re-evaluation are summarized in Table 3. A hiatal hernia was seen in 42.3 % (47/111) of the patients.

### Prevalence of registered IPF patients in Finland

The overall prevalence was 8.6 cases/100 000 (Table 4). The number of patients in relation to the local population as well as the participation rates was different between the university hospital cities (*p* < 0.001, *p* = 0.0208, *X*^2^-test respectively). In 2012, 17 patients of these 111 cases were newly diagnosed. Information on familial or sporadic IPF was available in 92/111 patients. Patient reported history (two or more IPF cases in the family) was used to identify familial form. Altogether six patients (6.5 % of the valid) had familial IPF.

**Table 4:** The population, number of patients included in the study, deaths, and prevalence 31 th December 31, 2012 in different regions

City	Population	IPF patients *n* (%)	Number of deaths (%)	Prevalence
Helsinki	603968	36 (32.4)	3/36 (8.3)	6.0
Turku	180225	17 (15.3)	2 /17(11.8)	9.4
Tampere	217421	21 (18.9)	1/21 (4.8)	9.7
Kuopio	105136	17 (15.3)	3/18 (16.7)	17.0
Oulu	190847	20 (18.0)	5/20 (25.0)	10.5
Total	1297597	111 (100)	14/111 (12.6)	8.6

# = Total population of the cities (Tilastokeskus, Statistic Finland 31 December 2012 [25]

### Deaths during 2012

Of the 111 study participants 14 died before the end of the year 2012. Of the 14 deceased patients 6 were women and 8 men. The mean age at death was 75.5 years. At diagnosis the mean FVC of predicted was 75.3 % and mean DLCO/VA of predicted 75.3 %. The median survival was 44.9 months after diagnosis. IPF was considered to be the immediate cause of death in 7 (50 %) of the cases. Pneumonia was the second most common immediate cause affecting 5 patients (36 %). Other two non-IPF-related immediate causes of death were intestinal strangulation and rupture of abdominal aortic aneurysm. IPF was considered to be underlying cause of death in 12 cases. In the two cases described above, the underlying cause of death was intestinal strangulation and aortic atherosclerosis.

### Smoking

Data on smoking was available in 98.2 % (109/111) of patients. Forty-four point one percent (44.1 %) were never-smokers, 45.9 % ex - and 8.1 % current smokers. On average, ex-smokers had an exposure of 27.3 (SD ± 16.4) pack-years and current smokers of 29 (SD ± 14.7) pack-years.

## Discussion

In this study we present baseline data of IPF patients in Finland. Our results indicate, that careful multidisciplinary evaluation of patients is necessary for obtaining accurate data on IPF epidemiology. Our results also show, that patients are diagnosed at a mild-moderate disease stage in Finland (over 50 % of patients have FVC above 80 %), which should be taken into account when drug reimbursement decisions are made in the future. Our study confirms that ICD-10 does not provide sufficient details for IPF diagnostics [18]. Other interstitial pulmonary diseases with fibrosis (J84.1) includes over 200 disease entities, which can lead to misclassification and overdiagnosis of patients if patients are not re-evaluated. In our study, as many as 70–80 % of the ICD-10 screened cases proved not to be IPF after clinical re-evaluation. As the awareness and classification of IIPs is getting more precise, it is extremely important to develop the diagnostic coding to meet the clinical needs. Our results contradict another recent study on IPF epidemiology [19] where the diagnostic code of ICD-9 were used as equivalent to IPF, and the results indicated a rising prevalence of IPF. When compared to a previous study from Finland [20], the prevalence seems in fact lower than 10 years before (16–18/100 000 [20] vs. 6.0–17.0/100 000).

The accuracy of the HRCT diagnosis in all the five university hospitals was shown to be high. In the radiological re-evaluation process, the presence of honeycombing was considered as one of the hardest things to define. Traction bronchiectasis and areas with combined emphysema and fibrosis can often be misread as honeycombing [21, 22], which may lead to overdiagnosis of IPF. On the other hand, mild honeycombing can be missed due to the conventional noncontiguous HRCT technique. The wider use of volumetric HRCT will probably ease the detection of honeycombing when multiplanar reconstructions from the thin slices will become available. The number of lung biopsies was low in our study 27/123 (22 %) possibly due to novel guidelines. After re-evaluation of histopathology, only one patient was excluded from our cohort suggesting that very few patients nowadays get a histopathological diagnosis, suggesting that the diagnostic accuracy of histopathology in tertiary hospitals is high.

### Geographical differences in IPF prevalence

In the UK, Navaratnam and colleagues have shown the incidence of IPF to vary regionally; it was highest in North West England [23]. Our results showed also regional differences and the highest prevalence of IPF was found in Eastern Finland, Kuopio. One explanation might be the high participation rate in Kuopio, which did not, however, account for the entire difference found. Smoking did not explain the high prevalence in Kuopio either, as the lowest numbers of smokers were found in Kuopio. In a previous Finnish study on sporadic and familial pulmonary fibrosis [20] it was suggested that familial IPF originated from Eastern Finland, from a cluster of multiplex families. Additionally, asbestos mining has taken place in the years 1904 – 1975 in a small town near Kuopio. The differences in IPF prevalence numbers between the university cities make the correct estimation of total number of patients more difficult. An ongoing study extending to local hospitals and the entire population will probably help in determining, whether there is a true geographical variation in the prevalence of IPF in Finland.

### Severity of IPF at diagnosis

According to our results, most IPF patients were diagnosed at a mild/moderate stage of the disease measured with lung function (FVC and DLCO at diagnosis). X-ray and spirometry are widely available in the Finnish primary health care centers, which could promote an earlier diagnosis. Nintedanib is not yet in use in Finland and pirfenidone treatment is reimbursed at the FVC range 50–80 %. Thus, over 50 % of our patients had lung function over the upper limit of this range. Our results warrant re-evaluation of the indications for drug therapy, when at best, the drug therapies only inhibit lung function decline. Indeed, the U.S. FDA recently approved pirfenidone for the treatment of idiopathic pulmonary fibrosis without limits of the disease severity and perhaps the indication in Europe will expand in the future. It is noteworthy that in our cohort, almost 10 % of the patients died within 3–4 years after diagnosis although they were diagnosed in mild-moderate functional stage and had most probably not received pirfenidone during their lifetime. Cigarette smoking is identified as a risk factor for IPF [13]. A study from Sweden showed that smoking has a dose-related association with increased risk of severe pulmonary fibrosis [24]. Table 5 shows some of the baseline data compared with published Danish and German cohorts [4, 14]. All studies show similar patient characteristics, but also differ in terms of the number of smokers and lung function. Results suggest that studies based on informed consent (such as InsightsIPF and FinnishIPF) in comparison to ie. The Danish study may yield slightly different patient cohorts in terms of disease severity, but do not rule out local differences in terms of diagnostics, risk factors or disease course. It would be interesting to compare these cohorts in terms of disease progression and mortality.

**Table 5:** Comparison of the baseline data from German, Finnish and Danish cohorts according to the published data

	Insights *N* = 502	FinnishIPF *N* = 111	Danish ILD *N* = 121
Mean age (years)	68.7	73.5	67.4
Male (%)	69	60	77
FVC % pred.	72.2	80.4	72
DLCO % pred.	35.5	57.3	42.3
Onset of symptom-Diagnosis (years)		1.9	2.3
Smokers current (ex-never) %	1 (60–39)	8 (46–44)	81^a^

^a^Current and ex-smokers

### Study limitations and strengths

There are several weaknesses in this study. In Finland, IPF is diagnosed almost exclusively at hospitals by respiratory physicians, which means that patients living in the university cities will attend the university hospitals. This, from our perspective, limits the disease identification-related bias to a minimum but it is still possible that some ILD cases have been misdiagnosed and, therefore, not codified as J84.1 or J84.9. The second limitation is that patients were included only by informed consent, ruling out patients that met the diagnostic criteria but were unable or unwilling to give informed consent. This approach was chosen partially because of the local legislation on patient data collection, but also to allow further contacts with patients if needed. This is a major limitation in our study, as the most advanced cases and rapid progressors are probably lost from the cohort. However, we have previously shown [1], that the published data on IPF varies according to the method used, so that any method used is going to yield in mere estimations of true prevalence.

The strength of this study is that all patient data were carefully re-evaluated according to the current guidelines. Only a small percentage of patients were excluded after the reassessment of the diagnostic HRCTs and surgical lung biopsies, which in part proves the high quality of the radiological and histopathological diagnostics of the public health care system. This study was, however, limited to university hospitals where the quality of diagnostics should be high and the results might not reflect the situation in smaller Finnish hospitals.

## Conclusions

Our results indicate that all patients coded with ICD-10 codes J84.1 or J84.9 should not be used in epidemiologic studies as equivalent to IPF. Only 20–30 % of these cases were IPF assessed on the basis of patient records. Histological and radiological re-evaluation still dropped out 12 patients out of these 123 patients. The nationwide prevalence of IPF can be estimated to be 8.6/100 000. In Finland IPF is diagnosed in mild-moderate stage, which with emerging drug treatments may lead to improved prognosis.

## Abbreviations

BMI: Body mass index
DLCO: Diffusing capacity of carbon monoxide
FEV1: Forced expiratory volume in 1 s
FVC: Forced vital capacity
HRCT: High-resolution computed tomography
ICD-10: International Classification of Diseases, 10th revision
ID: Identification number
IIP: Idiopathic interstitial pneumonia
IPF: Idiopathic pulmonary fibrosis
NSIP: Non-spesific interstitial pneumonia
UIP: Usual interstitial pneumonia
